# Use of algorithms for identifying patients in a German claims database: learnings from a lung cancer case

**DOI:** 10.1186/s12913-022-07982-8

**Published:** 2022-06-28

**Authors:** Sina Neugebauer, Frank Griesinger, Sabine Dippel, Stephanie Heidenreich, Nina Gruber, Detlef Chruscz, Sebastian Lempfert, Peter Kaskel

**Affiliations:** 1 MSD SHARP & DOHME GmbH, Levelingstrasse 4A, 81673 Munich, Germany; 2 Department of Hematology and Oncology, Internal Medicine-Oncology, Pius Hospital, Medical Campus University of Oldenburg, Cancer Center Oldenburg, Georgstrasse 12, 26121 Oldenburg, Germany; 3 Organon GmbH, Weystrasse 20, 6006 Lucerne, Switzerland; 4 CONVEMA Versorgungsmanagement GmbH, Karl-Marx-Allee 90A, 10243 Berlin, Germany; 5 HCSL Healthcare Consulting Sebastian Lempfert e.K., Bekwisch 32, 22848 Norderstedt, Germany; 6 MSD SHARP & DOHME GmbH (former address of MSD), Lindenplatz 1, 85540 Haar, Germany

**Keywords:** Lung cancer, ICD-10-GM classification, Real-world data (RWD), Cancer classification, NSCLC, SCLC

## Abstract

**Background:**

The analysis of statutory health insurance (SHI) data is a little-used approach for understanding treatment and care as well as resource use of lung cancer (LC) patients in Germany. The aims of this observational, retrospective, longitudinal analysis of structured data were to analyze the healthcare situation of LC patients in Germany based on routine data from SHI funds, to develop an algorithm that sheds light on LC types (non-small cell / NSCLC vs. small cell / SCLC), and to gain new knowledge to improve needs-based care.

**Methods:**

Anonymized billing data of approximately four million people with SHI were analyzed regarding ICD-10 (German modification), documented medical interventions based on the outpatient SHI Uniform Assessment Standard Tariff (EBM) or the inpatient Operations and Procedure Code (OPS), and the dispensing of prescription drugs to outpatients (ATC classification). The study included patients who were members of 64 SHI funds between Jan-1st, 2015 and Dec-31st, 2016 and who received the initial diagnosis of LC in 2015 and 2016.

**Results:**

The analysis shows that neither the cancer type nor the cancer stage can be unambiguously described by the ICD-10 coding. Furthermore, an assignment based on the prescribed medication provides only limited information: many of the drugs are either approved for both LC types or are used off-label, making it difficult to assign them to a specific LC type. Overall, 25% of the LC patients were unambiguously identifiable as NSCLC vs SCLC based on the ICD-10 code, the drug therapy, and the billing data.

**Conclusions:**

The current coding system appears to be of limited suitability for drawing conclusions about LC and therefore the SHI patient population. This makes it difficult to analyze the healthcare data with the aim of gathering new knowledge to improve needs-based care. The approach chosen for this study did not allow for development of a LC differentiation algorithm based on the available healthcare data. However, a better overview of patient specific needs could make it possible to modify the range of services provided by the SHI funds. From this perspective, it makes sense, in a first step, to refine the ICD-10 system to facilitate NSCLC vs. SCLC classification.

**Supplementary Information:**

The online version contains supplementary material available at 10.1186/s12913-022-07982-8.

## Background

Lung cancer is one of the most common causes of cancer-related death in both men and women in Germany [1–3]. In 2016 alone, lung cancer claimed the lives of 29,324 men and 16,481 women in Germany. According to the Federal Robert Koch Institute (Berlin), this corresponds to a standardized death rate of 45.7 for men and 22.6 for women [4].

A histological distinction is made between non-small cell lung cancer (NSCLC) and small cell lung cancer (SCLC) [1–3]. NSCLC dominates in terms of incidence, accounting for around 80% of all malignant lung tumors [1, 2, 5]. SCLC, by contrast, has a significantly lower incidence rate of 20% [1, 3, 6] but is prognostically even less favorable due to early lymph node and distant metastasis, which is also reflected in significantly lower survival rates compared to NSCLC [5, 6]. Since most lung cancers are usually only diagnosed at an advanced stage, prompt, targeted therapeutic or palliative intervention is required [7]. This poses a considerable challenge for therapists. This aspect, together with the large number of people affected, suggests that developing and ensuring adequate needs-based care is of paramount importance for patients.

In recent years the focus has increasingly been placed on the use of “real-world data” (RWD) [8–11]. RWD provide insights into the realities of daily practice as well as the patient population relevant to everyday life, including individual differences between patients. RWD also include data compiled in the healthcare databases of statutory health insurance (SHI) funds and health registers [12].

In Germany, the analysis of the healthcare data of SHI patients is used cautiously [9, 13], although it is a good way to model the routine care of patients. For example, the German variant of the ICD-10 coding of the World Health Organization (WHO) [14] (ICD-10-German Modification / GM) allows patients to be specifically identified on the basis of the coded clinical picture. The associated anonymized healthcare data can be consulted to analyze the use of medical interventions and prescription drugs in routine practice as described by aggregated data. Such a retrospective analysis of the healthcare situation of patients can be taken into account when shaping statutory healthcare policies. It can also form the basis for modifying statutory services. The use of health data from SHI funds is particularly useful in the case of high-prevalence clinical pictures, such as lung cancer, as it can help to identify options for improving the needs-based care of patients. Such was the aim of the observational, retrospective, longitudinal analysis of structured data presented here. It evaluated the healthcare data of SHI patients who were diagnosed with lung cancer in 2015 and 2016. Another goal was to develop an algorithm that allows conclusions to be drawn about the respective cancer types (NSCLC vs. SCLC) based on the billing data of SHI patients. This facilitates the acquisition of new knowledge about the diagnosis of lung cancer with the help of the SHI databases.

## Methods

### Data source

The present study analyzed the healthcare data of approximately four million insured patients from the research database of the Institute for Applied Health Research (InGef, formerly HRI) [15] in accordance with the legal requirements of Art. 287 of the German Social Security Code V (SGB V) and Art. 75 of the German Social Security Code X (SGB X) [16, 17] and the guidelines of good pharmacoepidemiological practice (GPP, 2008) [18]. The InGef research database contains anonymized data on around 6.7 million people living in Germany (corresponding to approximately 5% of the total number of insured people in Germany) who are members of 64 SHI funds [15]. These 64 SHI funds are mainly company and guild-based. The healthcare data of the SHI members are transferred directly by the healthcare service provider to a specialized data center belonging to the SHI funds [15]. One of the tasks of the data center is to anonymize the healthcare data before it is fed into the InGef research database. The data used in this analysis include the coded diagnoses according to ICD-10-GM [14] and the medical procedures according to the German outpatient Uniform Assessment Standard Tariff (EBM) [19] or the German inpatient Operations and Procedure Code (OPS) [20]. They also contain (outpatient) drug prescriptions according to the Anatomical Therapeutic Chemical Classification System (ATC) [21]. The analysis was based on a predefined analysis plan. Anonymized SHI data were used for this research and presented in aggregated form only. The use of such data is not subject to ethics committee approval in Germany. All InGef studies need approval from the internal InGef (formerly HRI) Scientific Research Group, which considers both ethical and scientific aspects of the intended study [15].

### Study design and analysis population

**Fig. 1:**
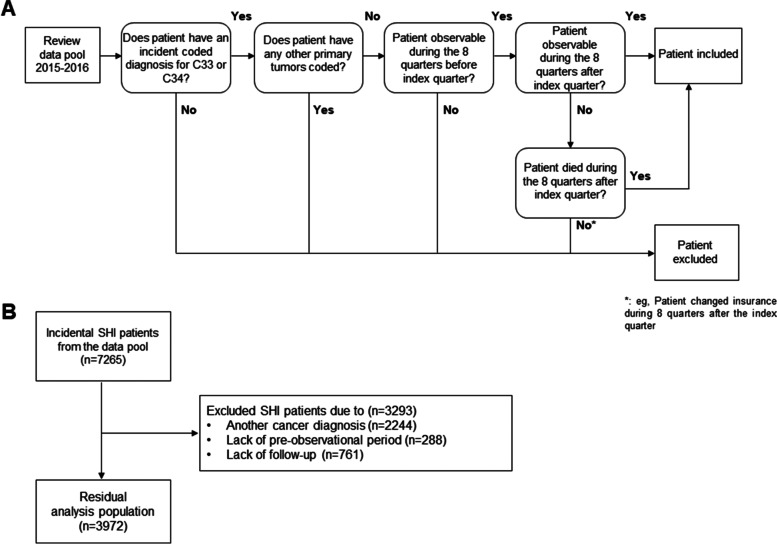
Study design and overview of the analysis population. **A** represents the algorithm used for selecting statutory health insurance (SHI) patients for the analysis. All SHI patients who received the initial diagnosis of lung cancer in 2015 or 2016 and for whom the cancer disease was coded as part of outpatient treatment in at least two different quarters of the observation period (= M2Q criterion) or for whom a confirmed diagnosis was coded once during inpatient treatment, which was regarded as confirmation of the diagnosis, were taken into account. The quarter in which the diagnosis of lung cancer was coded for the first time represents the index quarter. All SHI patients for whom other cancer diagnoses were documented were excluded from the analysis. **B** gives an overview of the total number of incidental SHI patients, the proportion of excluded SHI patients after application of the algorithm, and the final analysis population

This study was an observational, retrospective, non-comparative longitudinal data analysis based on anonymized routine data from 64 SHI funds on the healthcare situation of insured patients diagnosed with lung cancer. It analyzed the healthcare data of patients insured by one of the SHI funds between January 1, 2015 and December 31, 2016, and for whom the diagnosis of lung cancer (malignant neoplasm of the trachea (C33) or malignant neoplasm of the bronchi and lungs (C34) according to ICD-10-GM) was coded initially in a quarter in the years 2015 or 2016 (index quarter), and was confirmed by way of a standardized procedure. Patients were excluded from the analysis if they had no confirmed diagnosis of lung cancer in the index quarter or if they had a diagnosis of further primary solid tumors (C00-C75 without C33, C34, C44 and C61) in the eight quarters before or after the respective index quarter. Lung cancer patients were only regarded as newly diagnosed, if there was no lung cancer diagnosis (C33, C34) during the eight quarters before the index quarter. This also ensured that patients had not been diagnosed of lung cancer before, because in such case as per guideline a follow-up visit would have been documented. For evaluation of the treatment lines (first line treatment/second line treatment) and treatment groups, data were used from the time period starting with the index quarter in 2015/2016 to the year 2018. Figures 1A and B give an overview of the selection algorithm for the SHI patients and the inclusion and exclusion criteria applied.

### Code groups

For the analysis of epidemiological questions, coding groups were defined according to the disease stage (no metastases, lymph node metastases, distant metastases). For this purpose, all SHI patients were taken into account for whom cancer was coded during outpatient treatment in at least two different quarters of the observation period so that the diagnosis was considered to be “confirmed”, or for whom a confirmed diagnosis was coded once during inpatient treatment (see Fig. 1A). The cohort of SHI patients without metastases includes all patients who were coded with C33 (malignant neoplasm of the trachea) or C34 (malignant neoplasm of the bronchi and lungs) in the index quarter. The cohort without metastases includes UICC clinical stage IA/IB (version 7) [22, 23] and, in part, stage IIA/IIB. The cohort with lymph node metastases includes all SHI patients who, in addition to C33 or C34, were also assigned the code C77 (secondary and unspecified malignant neoplasm of the lymph nodes). These include clinical stages IIA/IIB (in part) and stages IIIA/IIIB. Patients who were assigned code C78 (secondary malignant neoplasm of the respiratory or digestive organs) or C79 (secondary malignant neoplasm at other sites not detailed) were assigned to the cohort with distant metastases (clinical stage IV). A distinction between squamous and non-squamous lung cancer is not yet possible based on the ICD-10-GM coding and was therefore not taken into account in the classification. In addition to the stages of the disease, demographic data such as age, gender, and region of residence were also recorded on the basis of the patients’ official municipality code [24] (urban vs. rural).

### Treatment groups

The patients were divided into treatment groups based on the drugs documented for them in first-line and, if applicable, second-line therapy [2, 3, 6]. First-line therapy was defined as the treatment whose commencement was coded in the index quarter itself or in the following quarter. Starting with the first treatment, all treatment-relevant billing data (based on drug prescriptions or on EBM and/or OPS) over a period of one month were collected and analyzed. Based on this, the patients were then assigned to one of the predefined treatment groups (Table 1). As for inpatient treatment, lung cancer therapies usually have an OPS code assigned in Germany. As not all drugs of interest have an assigned OPS code, in case of application of therapies with no OPS code assigned due to their generic status since decades, e.g., platinum derivates, OPS codes indicating chemotherapy were used, e.g., “non-complex chemotherapy” (OPS 8–542) or “moderate complex and intense block-chemotherapy” (OPS 8–543). By this process of elimination it was ensured that no patient coded as receiving inpatient treatment was missed [2, 3].

**Table 1:** Code algorithm for assigning insured patients to treatment groups and cancer type

Treatment group	Drug name	ATC	OPS	Cancer type
Immunotherapy	Cetuximab’	L01XC06	6–001.a	NSCLC
	Bevacizumab’	L01XC07	6–002.9	NSCLC
	Ipilimumab	L01XC11	6–006.h	NSCLC
	Nivolumab	L01XC17	6–008.m	NSCLC
	Pembrolizumab	L01XC18	6–009.3	NSCLC
	Necitumumab’	L01XC22	6–009.g	NSCLC
	Durvalumab	L01XC28		NSCLC
	Avelumab	L01XC31		NSCLC
	Atezolizumab	L01XC32		NSCLC
	Other immunotherapies		8–547	
Inhibitors’	Gefitinib	L01XE02		NSCLC
	Erlotinib	L01XE03		NSCLC
	Afatinib	L01XE13		NSCLC
	Crizotinib	L01XE16	6–006.c	NSCLC
	Dabrafenib	L01XE23	6–007.5	NSCLC
	Trametinib	L01XE25	6–009.7	NSCLC
	Ceritinib	L01XE28	6–008.a	NSCLC
	Nintedanib	L01XE31		NSCLC
	Osimertinib	L01XE35		NSCLC
Chemotherapy	Cyclophosphamide	L01AA01		SCLC
	Ifosfamide	L01AA06		SCLC
	Trofosfamide	L01AA07		SCLC
	Lomustine	L01AD02		SCLC
	Temozolomide	L01AX03	6–002.e	
	Temozolomide	L01AX03	6–005.c	
	Pemetrexed	L01BA04	6–001.c	NSCLC
	5-Fluoruracil	L01BC02		NSCLC
	Gemcitabine	L01BC05	6–001.1	NSCLC
	Capecitabine	L01BC06		
	Vinorelbine	L01CA04		NSCLC
	Etoposide	L01CB01		SCLC
	Nab-paclitaxel	L01CD01	6–005.d	NSCLC
	Paclitaxel	L01CD01	6–001.f	NSCLC
	Docetaxel	L01CD02	6–002.h	
	Doxorubicin	L01DB01	6–001.b	SCLC
	Doxorubicin	L01DB01	6–002.8	SCLC
	Epirubicin	L01DB03		SCLC
	Mitomycin	L01DC03		NSCLC
	Cisplatin	L01XA01		
	Carboplatin	L01XA02		SCLC
	Oxaliplatin	L01XA03		
	Topotecan	L01XX17	6–002.4	SCLC
	Irinotecan	L01XX19	6–001.3	SCLC
	Liposomal irinotecan	L01XX19	6–009.e	SCLC
	Folic acid	V03AF03		NSCLC
	Non-complex chemotherapy		8–542	
	Moderately complex and intensive chemotherapy cycle		8–543	
	Highly complex and intensive chemotherapy cycle		8–544	
	Hyperthermic chemotherapy		8–546	
	Percutaneous closed organ perfusion with chemotherapeutic agents		8–549	
Treatment group	**Definition**	**EBM**	**OPS**	
Radiotherapy	Inpatient radiotherapy		OPS 8–52	
	High-voltage therapy	EBM 25320 EBM 25321 EBM 25322 EBM 25323		
	Brachytherapy	EBM 25331 EBM 25333		
	Radiotherapy planning	EBM 25340 EBM 25341 EBM 25342		
Study^a^		EBM 13461 or EBM 13492 in conjunction with EBM 13494		
		EBM 02100 (at least 10 min) or EBM 02101 (at least 60 min)		
		EBM 01510 (2 h) EBM 01511 (4 h) EBM 01512 (6 h)		
Other treatment	No documentation of previous treatment in the index quarter, in the first month of first-line therapy or second-line therapy, no documentation of previous treatment in the first quarter after the index quarter or the start of second-line therapy OR inpatient treatment (OPS 8–541) within one month after the index quarter or in the first month after the start of second-line therapy or treatments within one quarter after the index quarter or in the first quarter after the start of second-line therapy			
Not observable in line	See definition of other treatment in the first section AND no follow-up documentation of the patient during first-line or second-line therapy			
Late therapy	See definition of other treatment in the first section AND possible follow-up documentation of the patient during first-line or second-line therapy AND Documentation of previous treatments after first-line or second-line therapy			
No follow-up diagnosis	See definition of other treatment in the first section AND possible follow-up documentation of the patient during first-line or second-line therapy AND no repeated documentation of the diagnosis of lung cancer after the index quarter or in another quarter (violation of IC1)			
Surgical intervention, no therapy	See definition of other treatment in the first section AND possible follow-up documentation of the patient during first-line or second-line therapy AND repeated documentation of the diagnosis of lung cancer after the index quarter or in another quarter (satisfaction of IC1) AND documentation of an operation within the observation phase of first-line or second-line therapy (Table 2)			
Bronchoscopy, no surgical intervention, no therapy	See definition of other treatment in the first section AND possible follow-up documentation of the patient during first-line or second-line therapy AND repeated documentation of the diagnosis of lung cancer after the index quarter or in another quarter (satisfaction of IC1) AND no documentation of an operation within the observation phase of first-line or second-line therapy (Table 2) AND documentation of bronchoscopy within the observation phase of first-line or second-line therapy			
No bronchoscopy, no surgical intervention, no therapy	See definition of other treatment in the first section AND possible follow-up documentation of the patient during first-line or second-line therapy AND repeated documentation of the diagnosis of lung cancer after the index quarter or in another quarter (satisfaction of IC1) AND no documentation of an operation within the observation phase of first-line or second-line therapy (Table 2) AND No documentation of bronchoscopy within the observation phase of first-line or second-line therapy			

*ATC* Anatomical Therapeutic Chemical Classification System, *EBM* German outpatient Uniform Assessment Standard Tariff, *NSCLC* Non-small cell lung cancer, *OPS* German inpatient Operations and Procedure Code, *SCLC* Small cell lung cancer

^a^Patients in this group did not receive any higher-priority treatment within the index quarter or the first month after the start of second-line therapy. Only supplementary services were billed here – not the actual underlying service (e.g. administration of a drug**).** This category was added, because, pursuant to Art. 35c (2) SGB V [25], in (academic) studies the statutory health insurance fund provides the standard services that are also provided in connection with prescription-only medication

Bevacizumab (targeting VEGF receptor), cetuximab (targeting EGF receptor), and necitumumab (targeting EGF receptor) are listed under the immunotherapy section because these are monoclonal antibodies

For the analysis of second-line therapy used, all cost items that were documented as therapeutic measures according to ATC, EBM or OPS were recorded again from one month after the start of first-line treatment. The one-month period from the time a prescription was first identified that did not correspond to the first-line therapy was analyzed, used for reassigning the patients to treatment groups, and defined as second-line therapy for the respective patient. It was possible for patients to remain in the same treatment group. Table 1 gives an overview of the first and second-line therapy groups.

### Cancer differentiation based on the drug therapy used

ICD-10-GM coding does not allow the SHI patients to be assigned to one of the cancer-type categories (NSCLC, SCLC). An attempt was therefore made to assign the patients to the respective cancer-type groups with the help of the therapy documented in the SHI billing data. This was based on the assumption that most of the drugs used to treat lung cancer are only approved for one of the two cancer types (approval status 2019). If the first-line therapy did not allow unambiguous assignment of the patient, the second-line therapy (if available) was also used to determine the cancer type. Patients for whom none of the drugs listed in Table 1 were documented could not be assigned to either of the two cancer types and were therefore placed in the group of unclassifiable patients (see also Supplemental file 1).

Patients for whom one of the listed drugs was documented but was not used in either the index quarter or the first month after the start of first-line therapy or in the first month after the start of second-line therapy were also regarded as unclassifiable.

### Surgical interventions

In order to identify the SHI patients who received a surgical intervention, the analysis considered the index quarter and the first quarter of first-line therapy. For OPS codes used to identify those patients who underwent a surgical intervention see Supplemental file 2.

### Therapists

The study was also designed to identify the institution primarily responsible for the treatment. The “therapist” was defined as the first physician to code the diagnosis of “lung cancer” at the relevant diagnostic stage. For this purpose, it was determined where the diagnosis was documented in the index quarter, in the quarter in which first-line therapy commenced and in the quarter in which second-line therapy commenced. A distinction was made between the following therapist types: inpatient hospital stay, outpatient hospital stay, other physician (i.e., oncologist, pulmonologist, radiologist, internist, primary-care physician, other office-based physician). The relevant codes are provided in Supplemental file 3. If several therapists were documented in the index quarter, a hierarchy analogous to the order shown in Supplemental file 3 was applied to define the therapist.

### Diagnostic measures

#### Biomarkers

Testing for lung-cancer-specific biomarkers is an important diagnostic criterion for the treatment of lung cancer. The study therefore analyzed the extent to which biomarker testing was billed for these patients with coded lung cancer. In addition, it was determined whether the testing occurred mainly during an inpatient hospital stay or as part of outpatient care. Billing codes EBM 19320, EBM 19321, and EBM 19322 and keys OPS 1–991 × and OPS 1–992 × in the index quarter and in the first month of the first-line therapy were used. Biomarker testing during this period was assigned to first-line therapy, while all biomarker tests that were documented from one month after the start of first-line therapy to one month after the start of second-line therapy were assigned to second-line therapy. Biomarker testing was considered to have been performed on an inpatient basis if the patient had a documented hospital stay in the two weeks prior to the test. Otherwise the test was regarded as an outpatient procedure because ambulatory care of outpatients can also be coded with inpatient OPS codes e.g., if bronchoscopy was performed in a hospital day clinic.

#### Bronchoscopy

The study also analyzed the extent to which diagnostic bronchoscopy was billed for lung cancer patients depending on the cancer type and the treatment used and whether it was performed on an outpatient or inpatient basis. For this purpose, the following codes were taken into account in the index quarter and in the first month of first-line therapy: OPS 1–690.0, OPS 1–690.1, OPS 1–620, EBM 09315 and EBM 13662. The distinction between inpatient or outpatient bronchoscopy was made in the same way as done for biomarker testing.

### Disease- and treatment-related impairment of freedom (time expenditure)

In this context, disease- and treatment-related impairment of freedom relates to a patient’s freedom in shaping their own lives (in contradistinction to the legal term of impairment of freedom in decision-making [Art. 4 of the German Civil Code (BGB) (2)] [26]), insofar as this can be modeled as time expenditure on the basis of the available data. Such disease- and treatment-related impairment of freedom was determined in order to quantify the coordinative and organizational effort imposed on the patient by the diagnosis and treatment of lung cancer coded for the first time. This was determined in relation to the cancer type and the region of residence. To this end, the four quarters before the index quarter, the index quarter itself, and up to eight quarters after the index quarter were analyzed. The number of visits to a pharmacy to obtain the necessary medications and the number of visits to a primary-care physician or specialist were also taken into account. For inpatient and outpatient hospital stays, the number of days spent in hospital were included. Patients with second-line therapy or without therapy, patients who could not be observed in line, and SHI patients whose cancer disease was not coded in the context of outpatient treatment in at least two different quarters were excluded from the analysis of disease- and treatment-related impairment of freedom.

### Statistical analyses

The data analysis had not relied on preliminary work by others and was carried out retrospectively and purely descriptively on the basis of a predefined analytical plan. The demographic data and the results of the analysis points were expressed in terms of number (n) and percentage (%). The breakdown was based on the cancer types to which the patients were assigned according to the treatment data. The patients’ demographic data included age, gender, and regional area. The age categories were divided into ≤ 60 years vs. > 60 years. The anonymized data were analyzed and presented in aggregated form using the SAS statistics program version 9.4 [27].

## Results

### Patient demographics

A total of 7265 SHI patients with lung cancer first documented in 2015 or 2016 were identified. Of those, 3293 were excluded from the actual analysis due to other documented cancer diagnoses (*n* = 2244, 30.9%) or a lack of pre-observation time (*n* = 288, 4.0%) or post-observation time (*n* = 761, 10.5%). In the end, the analysis population comprised 3972 patients (54.7%) (Fig. 1B). The Federal Center for Cancer Registry Data of the Robert Koch Institute (Berlin) reports a standardized incidence rate of 31.9 (women) and 59.9 (men) per 100,000 population for 2015 (age-standardized according to the age of the European population). By way of comparison, the analysis population reflects the real situation in Germany very well.

Differentiation of cancer types on the basis of the ATC classification and the OPS codes was only possible for a quarter of the patients (*n* = 1033, 26.0%). Of those, 780 (19.6%) were assigned to the NSCLC cohort and 253 (6.4%) to the SCLC cohort. No classification based on the therapy received was possible for 2939 patients (74.0%) (Supplemental file 1B).

Most of the patients who could be evaluated (total *n* = 3922) were ≥ 60 years old (*n* = 3031, 77.3%), male (*n* = 2502, 63.0%), and residents of an urban region (*n* = 3128, 78.9%).

**Fig. 2:**
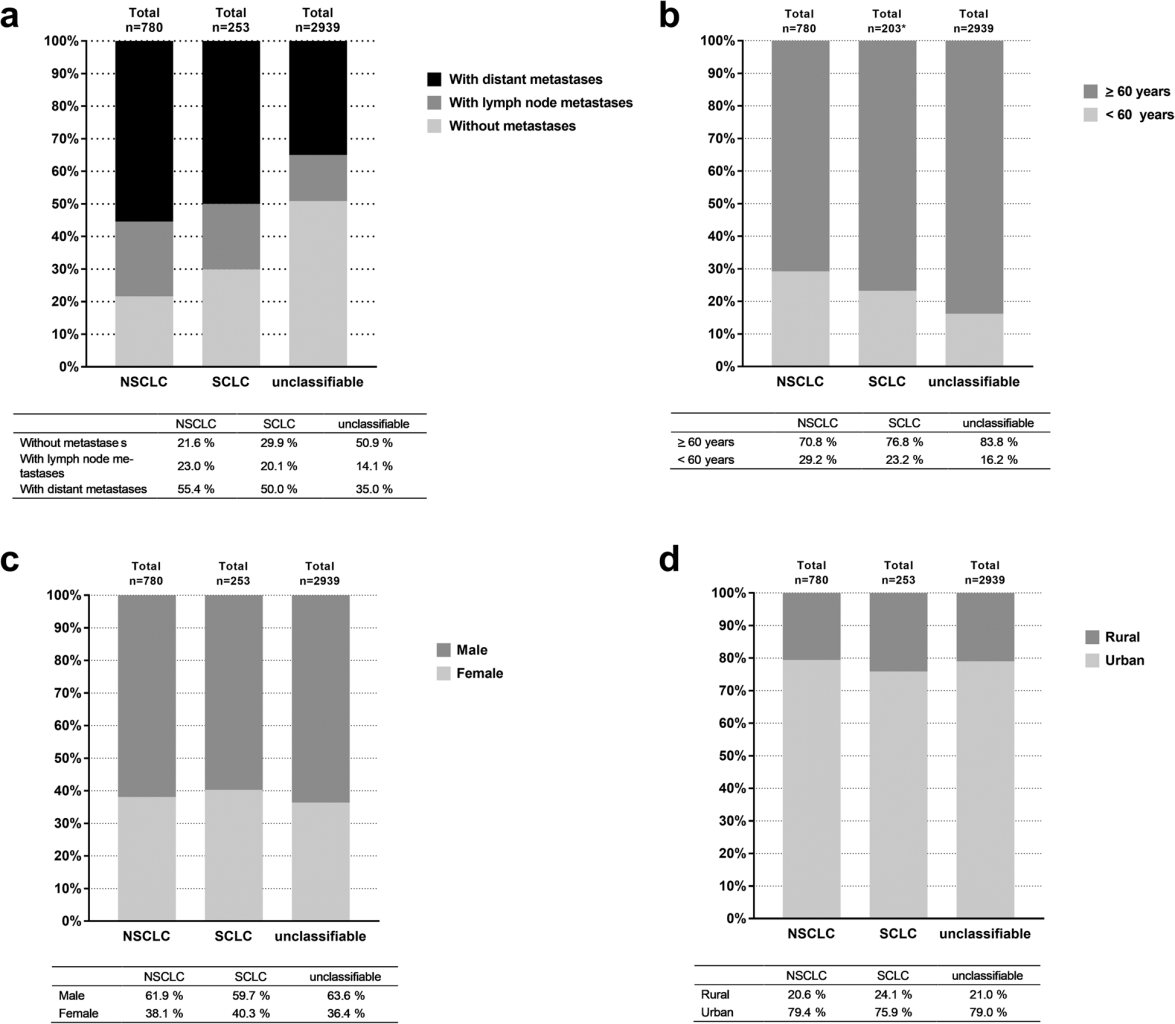
Patient demographics. Cancer stages (**A**), age structure (**B**), gender ratio (**C**), and geographical distribution (**D**) of the analysis population are shown in relation to the cancer type (NSCLC, SCLC) and of the group of unclassifiable patients. The percentage expresses the proportion of patients who exhibited the respective characteristics within each cancer type. *: Age could not be evaluated for 50 patients

**Fig. 3:**
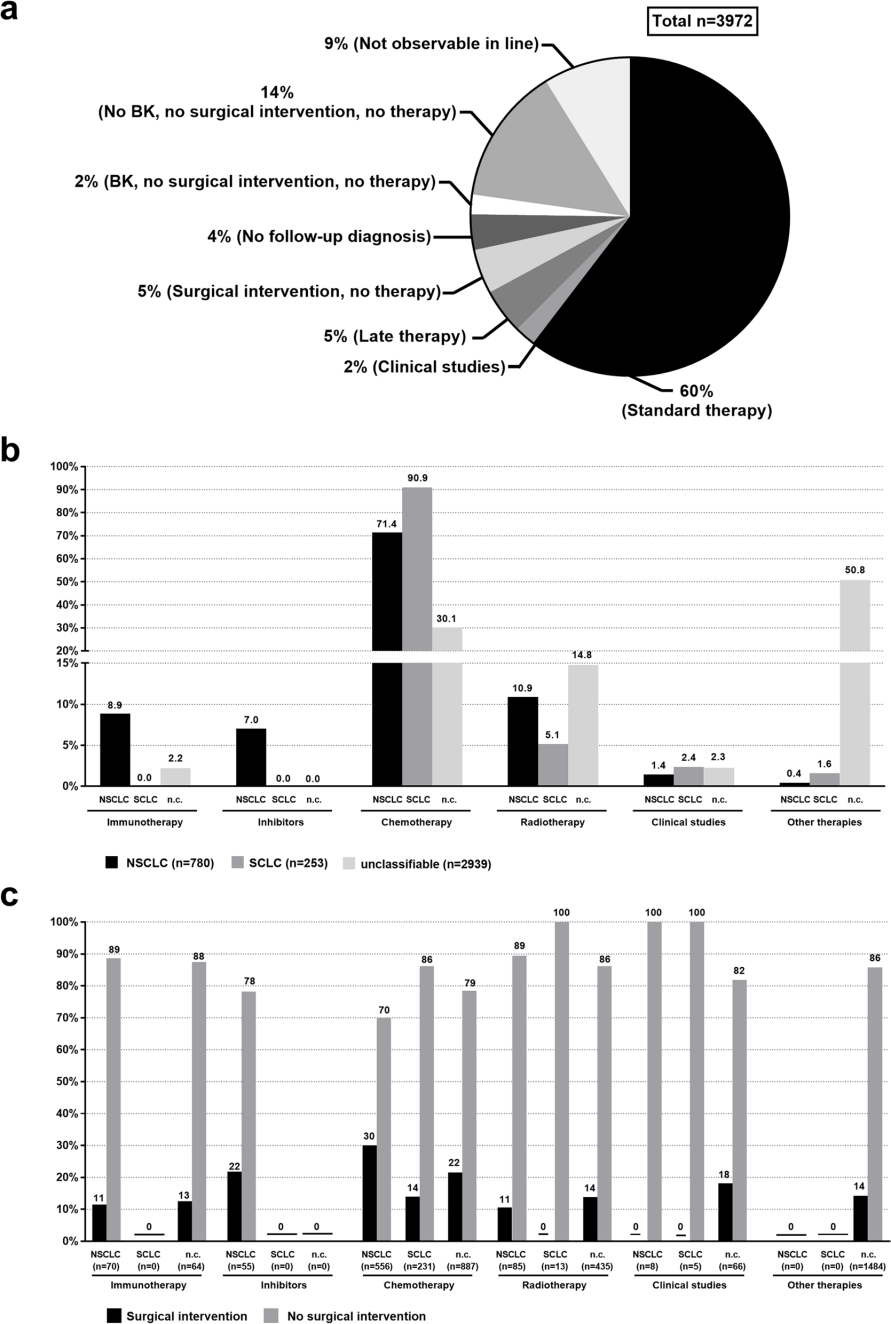
Coded treatments. **A** gives an overview of the therapeutic measures in the overall analysis population, including those patients who could not be observed in line, for whom none of the defined therapies were documented, or who received second-line therapy (late therapy). The proportion of the total population who received or did not receive the respective therapy and/or measure is expressed in percent. **B** Comparison of the use of drug therapy and radiotherapy in relation to the classification group. The percent figures indicate the proportion of patients within the respective cancer-type population for whom the listed therapy was coded. n indicates the number of patients who were assigned to each cancer type or the group of unclassifiable patients. The patients were assigned to the respective therapies hierarchically. **C** Percent of patients for whom a surgical intervention was coded, using the cancer type and the coded first-line therapy. n indicates the number of patients who were assigned both to the respective cancer type and to the listed therapy based on the coding. BK: bronchoscopy, n.c.: not classifiable

Considering the individual cohorts, it is noteworthy that around a quarter of the patients who could be assigned were younger than 60 years (NSCLC: 29.3%, SCLC: 23.2%), while only 16.2% of those who could not be classified fell into the < 60 years age group (Fig. 2B). Overall, the median age at diagnosis was 65 years (min. 35, max. 88) for the NSCLC cohort and 66 years (min. 42, max. 87) for the SCLC cohort. For the group of unclassifiable patients, the median age was 71 years (min 17, max 98). With regard to age, all three cohorts (NSCLC, SCLC, unclassifiable) contained more men over 60 (2079 men vs. 1093 women in total; Fig. 2C). By contrast, the gender ratio appeared balanced among those under 60 (423 men vs. 349 women, data not explicitly shown). The analysis of urban vs. rural distribution based on the official community code (24) showed little difference between the NSCLC patients (79.4% [*n* = 617] vs. 20.6% [*n* = 160], urban vs. rural) and the unclassifiable patients (79.0% [*n* = 2319] vs. 21.0% [*n* = 618], urban vs. rural; Fig. 2D). In the SCLC group, on the other hand, the proportion of patients in rural areas was somewhat larger (24.1%, *n* = 61; Fig. 3D). For both the NSCLC patients and the SCLC patients the diagnosis was made at an advanced stage (78.3% and 70.4%, respectively) (Fig. 2A). By contrast, the initial diagnosis of unclassifiable patients was coded in the early stages without the presence of metastases in half of the patients (50.9%).

### Treatment groups

The analysis shows that 60% of the SHI patients analyzed received one of the standard therapies during first-line therapy (immunotherapy, therapy with inhibitors, chemotherapy, radiotherapy, Fig. 3A, see Table 1 for definitions). 2% of the patients were enrolled in studies during first-line therapy, and 5% received late therapy (see Table 1 for definition). A surgical intervention was coded for 5% of the analysis population, but there was no documentation of further therapeutic measures. For 20% of the patients, only the initial diagnosis was made without any further measures.

Irrespective of which of the two cancer types the patients were assigned to, the majority of those for whom therapy was documented received chemotherapy (NSCLC: 71.4%, SCLC: 90.9%, Fig. 3B). Among the unclassifiable patients, only 30.1% received chemotherapy. Of those with NSCLC, 15.9% were treated by immunotherapy or inhibitor therapy, while 10.9% only received radiotherapy. In the group of unclassifiable insured persons, around half (50.8%) did not receive any of the defined therapies.

In the group of patients with NSCLC, 30.0% of those who received chemotherapy were billed for surgery in the index quarter or in the following quarter (Fig. 3C). Surgical interventions were also documented for this period for NSCLC patients who were billed for immunotherapy or inhibitor therapy. In this case the percentage was higher among patients who received inhibitor therapy than among those who received immunotherapy (21.8% vs. 11.4%). In the SCLC group, chemotherapy was billed for most of the patients (90.9% of all patients with SCLC). The proportion of those who also had a documented surgical intervention was 13.9%. Overall, fewer surgical interventions were documented among the SCLC patients than among those with NSCLC.

In the context of second-line therapy, surgical interventions were only coded for those patients in the NSCLC (3.3%) and unclassifiable (1.8%) cancer-type groups. However, the proportion of patients with surgical interventions was very low in both classification groups and significantly lower than in the first-line therapy (for details see Supplemental file 4).

### Therapists

For the majority of all patients in the analysis population, the initial diagnosis was coded during hospitalization. At 74.3%, the proportion in the group of patients with SCLC was smaller than in the group of patients with NSCLC (81.2%) and in the group of unclassifiable patients (81.8%, Table 2). Interestingly, the proportion of patients with SCLC whose initial diagnosis was coded primarily in the outpatient setting was greater than that of patients with NSCLC and the unclassifiable patients. If the cancer types are examined in detail in relation to the therapy used, we see that most patients with NSCLC were diagnosed as inpatients regardless of the therapy. Only in the case of patients who received inhibitor therapy were around 63.6% initially coded with the diagnosis of lung cancer during an inpatient hospital stay. For the other therapies, it was at least 72.7% of the patients. For patients in the NSCLC group treated with inhibitors, the hospital documented outpatient treatment in 12.7% of cases. For patients in the SCLC group, the coding of the initial diagnosis was also mainly carried out in the hospital (proportion of each treatment group at least 72.6%). In the case of unclassifiable patients, the hospital was also the most common “therapist” for all treatment groups (proportion for each treatment group at least 54.5%).

**Table 2:** Treating institution or physician. The table shows the institution or physician that coded the diagnosis for each classification group. The percent figures express the proportion of patients coded by each “therapist” category. A hierarchy was defined to avoid patients being assigned to multiple categories (see also Supplemental file 3)

%	Hospital inpatient	Hospital outpatient	Other physician	Unknown	Total
NSCLC	**81.2**	**4.7**	**9.9**	**2.2**	**100.0** **(98.0)**
SCLC	**74.3**	**5.1**	**18.2**	**0.0**	**100.0** **(97.6)**
Unclassifiable	**81.8**	**2.9**	**12.1**	**1.5**	**100.0** **(98.3)**

For second-line therapy, as already observed for first-line therapy, the diagnosis was coded for most of the patients during an inpatient stay in hospital (NSCLC: 69.9%; SCLC: 64.0%, unclassifiable: 72.9%). It was noticeable that the proportion of patients with SCLC who were documented during an outpatient hospital stay was significantly higher at 11.1% than the proportion for whom this applied with regard to first-line therapy (for details see Supplemental file 5).

### Diagnostic measures

**Fig. 4:**
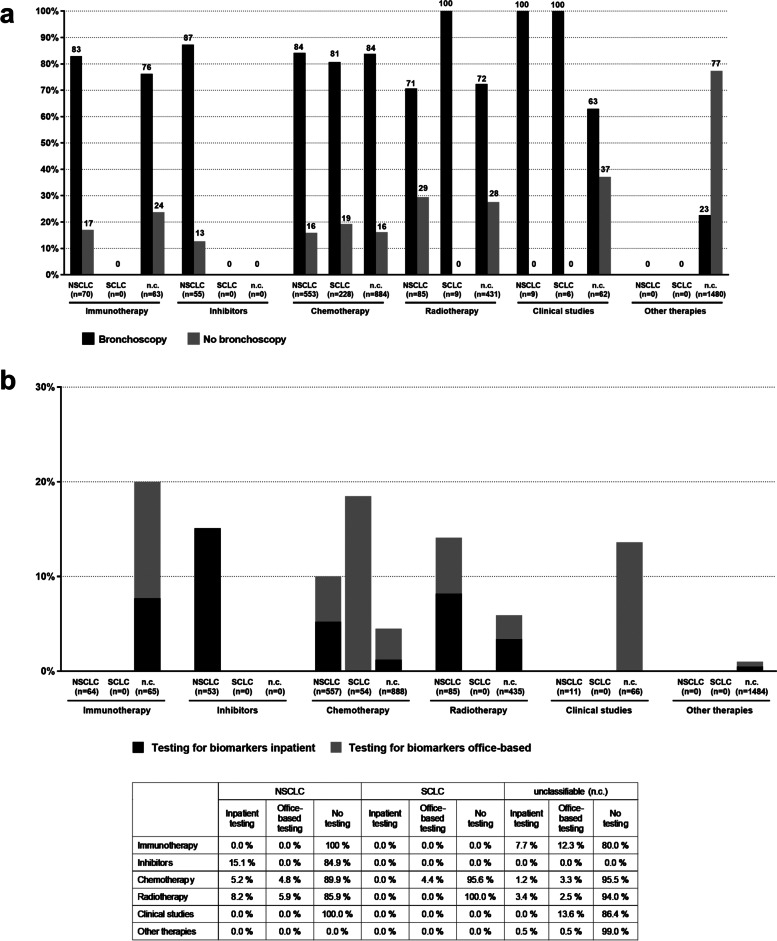
Diagnostic measures used. The diagnostic measures “bronchoscopy” (**A**) and “testing for biomarkers” (**B**) were analyzed concerning cancer type and the coded first-line therapy. **A** shows the percent of patients who had bronchoscopy (black bar, ■) or who did not have bronchoscopy (white bar, □) in relation to the coded first-line therapy within the cancer type (100% = all patients of the treatment group within the cancer classification group). **B** lists the biomarker testing of the incidental patients. This shows the percent of patients for whom coded biomarker testing was carried out in connection with standard care within the treatment group of each cancer classification group (*n* = 100%), taking into account the respective coding therapist (Inpatient vs. other office-based testing) vs. no biomarker testing). The percent figures are listed in the table below the graphic

It was found that all the bronchoscopies were documented in an inpatient setting. Bronchoscopy was billed for the majority of both NSCLC and SCLC patients (NSCLC: 82.7%, SCLC: 80.8%). By contrast, no more than 68% of the unclassifiable patients underwent bronchoscopy (Fig. 4A). The unclassifiable patients who received other treatments were the only group in which the proportion of patients without bronchoscopy was significantly higher than the proportion of those with bronchoscopy (77.3% vs. 22.7%).

In second-line therapy, bronchoscopy was documented for only 9.6% of the patients with NSCLC, in no cases for patients with SCLC, and for 5.5% of unclassifiable patients (for details see Supplemental file 6).

Analysis of the billing data for lung-cancer-specific biomarker testing revealed that SHI was charged only for 10.8% of the NSCLC patients and 5.2% of the SCLC patients based on EBM. 42.8% of the biomarker tests were charged in the hospital setting and 57.2% by other doctors. Among the NSCLC patients, the proportion of those accounted for in the “inhibitor therapy” treatment group was highest (15.1%), followed by those who received radiotherapy (14.1%, Fig. 4B). According to the EBM only 4.4% of the SCLC patients were accounted for, all of whom were in the chemotherapy treatment group. In relation to the total number of unclassified insured persons and without reference to the treatment groups, only 3.5% of the unclassifiable patients were accounted for biomarkers. Interestingly, charging for no biomarker testing was documented for the “study” treatment group for either NSCLC or SCLC patients, whereas 13.6% of the unclassifiable patients in the “studies” treatment group were tested. It needs to be emphasized here that no data from integrated healthcare contracts (charged separately from EBM) were analyzed, for which reason this is probably a gross underestimate.

In the second-line therapy, biomarker testing was billed for only 1.2% of the patients included in the analysis (*n* = 3966) (see Supplemental file 6 for details). No biomarker tests were billed for patients in the SCLC group during second-line therapy.

### Disease- and treatment-related impairment of freedom

**Fig. 5:**
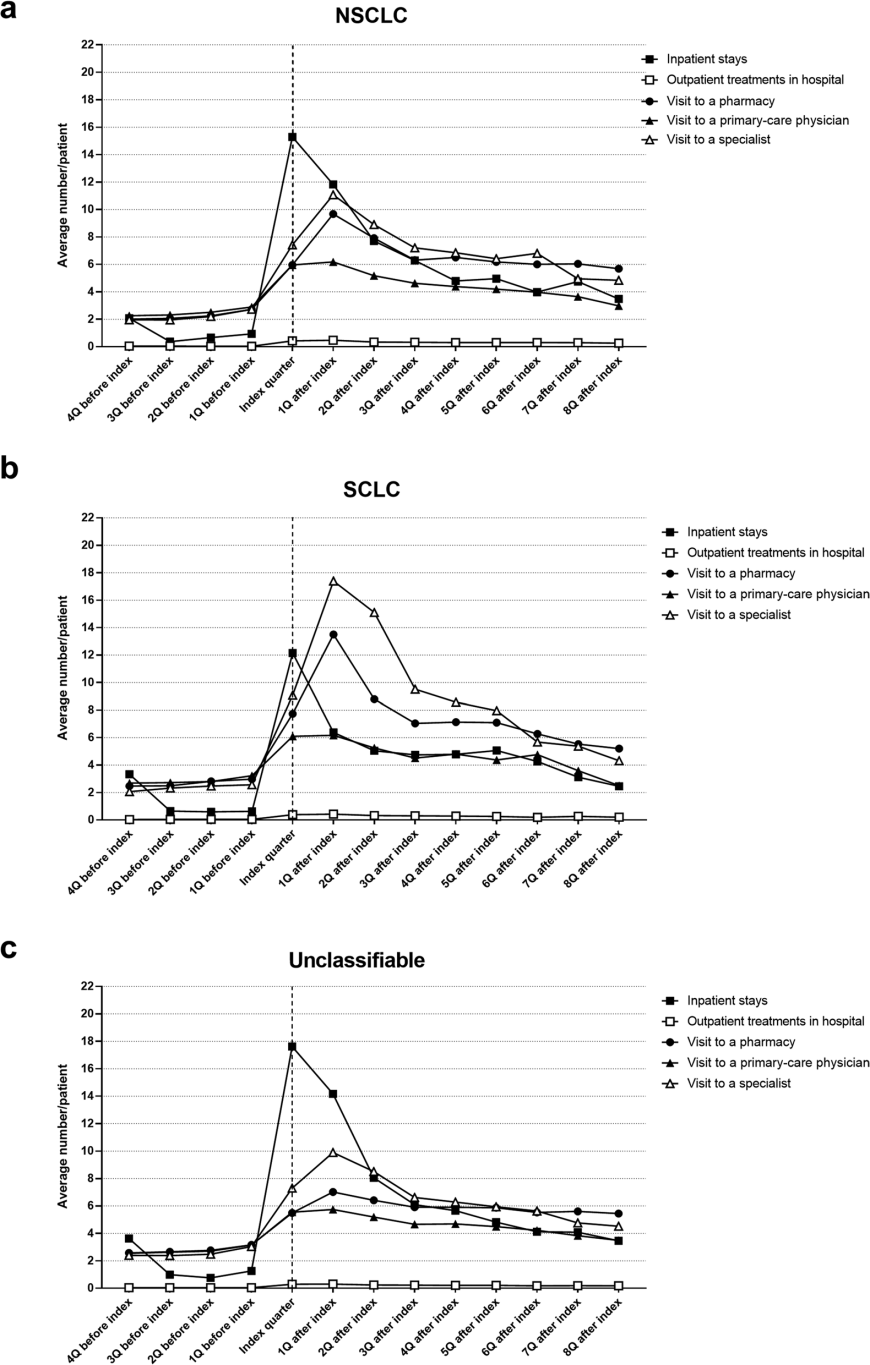
Disease- and treatment-related impairment of freedom in relation to the cancer type. Disease- and treatment-related impairment of freedom are shown based on the time expenditure in each classification group due to the number of days required for inpatient stays (- ■ -), the number of outpatient treatments in hospital (- □ -), the number of visits to a pharmacy (- • -), and the number of visits to a primary-care physician (- ▲ -) or specialist (- Δ -) beginning four quarters (4Q) before and ending eight quarters (8Q) after the index quarter. All visits by the patients within a cancer classification group were taken into account. **A** shows the time expenditure of the NSCLC patients due to illness and treatment, (**B**) that of the SCLC patients, and (**C**) of the unclassifiable incidental patients. The index quarter is indicated by the dashed vertical line (-—-). Q: quarter

An important determinant for the well-being of the patients is the time expenditure due to illness and treatment. It was found that this was greatest in the index quarter and in the quarter after the index quarter for all cancer types, although there were clear differences between the individual cancer types (Fig. 5). The number of inpatient stays was significantly higher for the NSCLC patients (mean number per patient: 15.3) and unclassifiable patients (mean number per patient: 17.6) than for the SCLC patients (mean number per patient: 12.3; Fig. 5). In the latter groups, the time expenditure due to illness and treatment was due mainly to visits to a specialist (NSCLC vs. SCLC vs. unclassifiable: 11.1 vs. 17.4 vs. 9.9) and a pharmacy (NSCLC vs. SCLC vs. unclassifiable: 9.7 vs. 13.5 vs. 7.0). The latter reached their numerical peak in the quarter following the index quarter in all three groups (Fig. 5).

### Urban–rural distribution of the patients

**Fig. 6:**
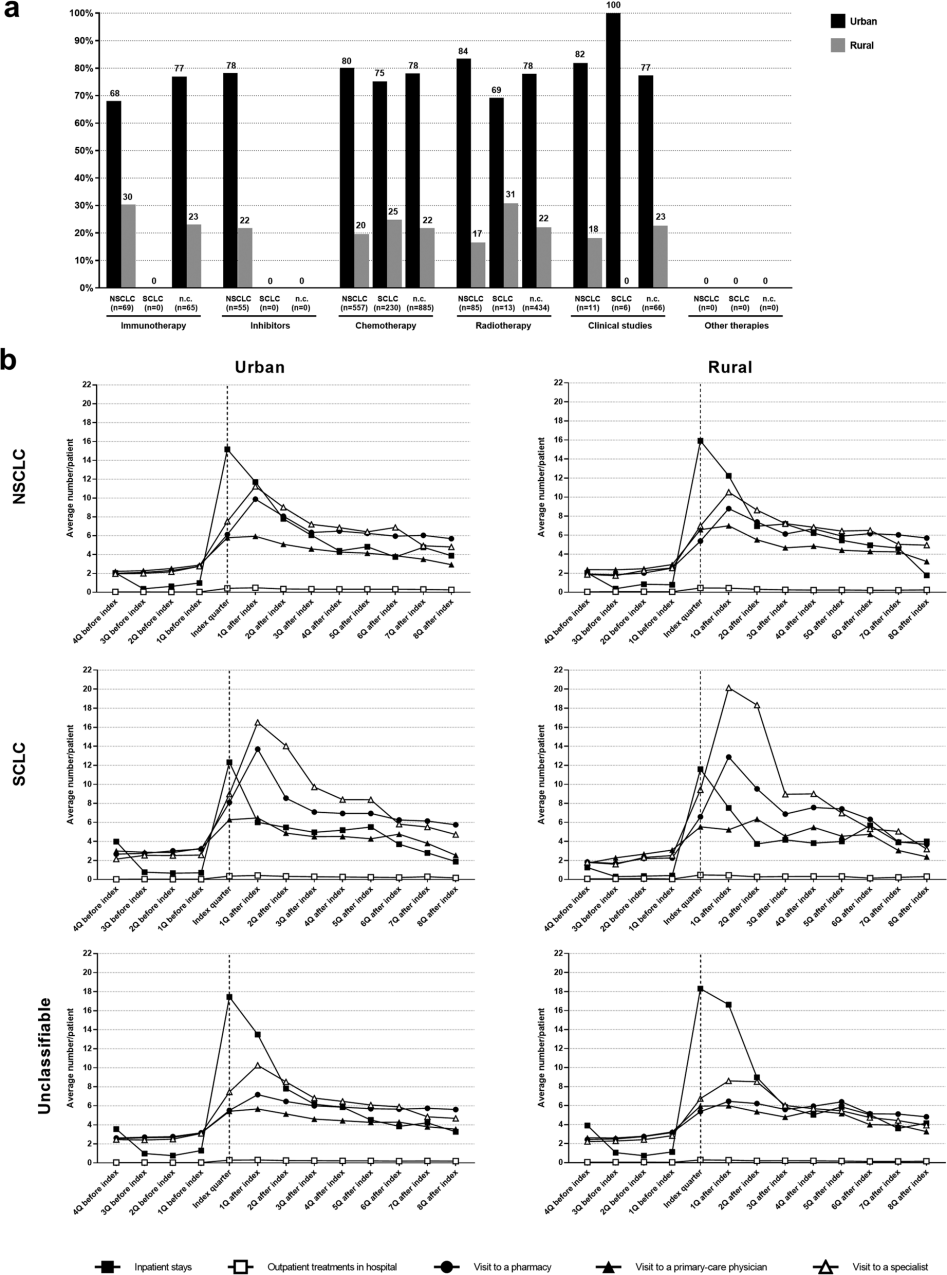
Influence of geographical distribution: urban vs. rural. **A** shows the coded therapy in relation to the cancer classification, considering the regional distribution of the patients (urban [black bars] vs. rural [gray bars]). In (**B**), the time expenditure due to illness and treatment is analogous to Fig. 5 but broken down by cancer classification groups and location (urban vs. rural). The time expenditure due to illness and treatment is defined by the days required for inpatient stays (- ■ -) as well as the number of outpatient treatments in a hospital (- □ -), the number of visits to a pharmacy (- • -), and the number of visits to a primary-care physician (- ▲ -) or specialist (- Δ -). The time frame spans the four quarters (4Q) before and eight quarters (8Q) after the index quarter. Q: quarter

Since urban areas have a higher density of physicians and hospitals, the question arose as to whether this has an impact on the treatment used or the time spent by patients as a result of illness and treatment. The analysis showed that in all three classification groups the proportion of urban patients in the respective treatment groups was greater than the proportion of rural patients (Fig. 6A). In relation to the individual classification groups, it was found that the mean number of days in hospital for the NSCLC patients was almost the same for both residential environments during the index quarter (urban vs. rural: 15.2 vs. 15.9). A similar picture emerged for the SCLC patients and the unclassifiable patients (SCLC: 12.3 vs. 11.6, unclassifiable: 17.4 vs. 18.3, in each case urban vs. rural, Fig. 6B). The aforementioned increase in the mean number of specialist and pharmacy visits in the quarter following the index quarter was observed in all three coding groups. It was noteworthy that it was highest in the group of SCLC patients (mean number of specialist visits in urban areas: 16.5, in rural areas: 20.2). By contrast, the mean number of specialist visits for the NSCLC patients was significantly lower at 11.2 (urban) and 10.5 (rural). The same applies to the unclassifiable patients at 10.3 (urban) and 8.6 (rural). The mean number of pharmacy visits was also significantly lower at 9.9 (NSCLC, urban), 8.8 (NSCLC, rural), 7.2 (unclassifiable, urban) and 6.5 (unclassifiable, rural) than for patients with SCLC [13.7 (urban), 12.8 (rural)], although there were only marginal differences between the urban and rural populations in all three patient groups. Overall, there were only minor differences between patients in urban areas and those in rural areas.

## Discussion

The present analysis served to retrospectively evaluate the healthcare situation of patients with statutory health insurance diagnosed with lung cancer in Germany on the basis of billing data from 64 statutory health insurance funds. The aim was to gain new knowledge for improved needs-based care delivery. The external validity of the database used was proven based upon a comparison to the German population as previously shown in terms of morbidity, mortality and drug usage. The database population used was slightly younger than the German population. Also, the proportion of members living in the eastern part of Germany was lower in the database used [15]. Targeted analyzes of the healthcare data on the extent of the relevant disease, the treatment strategies, and the use of new treatment methods, as well as the patients’ impairment of freedom due to illness and treatment provide a way to model the real-world routine clinical situation [11]. In the context of the German healthcare system, the hitherto rare analysis [13] of healthcare databases can help to identify and specify needs-based care services. The results of the analysis can also serve as a basis for discussing the healthcare services required for the diagnosis and treatment of lung cancer. An algorithm is required to specifically filter out the relevant patients within a healthcare database and thus permit a retrospective analysis and evaluation of the healthcare situation of large patient groups. One of the aims of the present analysis was therefore to establish this approach taking lung cancer as an example. Based on the ICD-10-GM coding as well as the EBM or OPS code, and the ATC classification of prescription drugs, which were documented by the therapist for billing purposes, it should be possible to draw conclusions about the cancer type present, as this is of crucial relevance to the type of treatment, the patient’s needs, and the further course of treatment. However, it turned out that at present, such assignment is only possible on basis of the healthcare data to a limited degree. Using the gold standard as cancer registries or electronic medical charts, it may be possible to develop an algorithm with high accuracy, but there was no gold standard for this study. Only a quarter of the total population could be reliably assigned in this way (NSCLC patients: 19.6%, SCLC patients: 6.4% of the total population). By contrast, an algorithm was developed for NSCLC patients in the USA which is based on clinical parameters [28]. It was therefore originally assumed that a similar algorithm to be developed de novo for the German health service context could also be applied to a German healthcare database. That was not the case. Reasons why this was not possible may be due to differences in coding practice in Germany. Although both, outpatient and inpatient treatment was observable in the data, there are no codes for laboratory examinations, and in contrast to the US, it is not possible to match the data with registries. Moreover, elderly persons might be less precisely diagnosed, and the treatment options may be more limited due to comorbidities and their general age-related health status [29, 30]. Further analyses in this area are required in order to identify and validate suitable variables of a filter algorithm. Current efforts to link healthcare and research may help to find better answers [31].

With regard to the treatment of the patients, most received standard therapy. It was noteworthy, however, that chemotherapy was used during first-line therapy for both NSCLC patients and SCLC patients (NSCLC patients: 71.4%, SCLC patients: 90.9%). Based on the current approval status, it was expected that the patients could be assigned to one of the two cancer types based on the coded drugs. For example, the treatment of SCLC relies on a platinum-based combination therapy with concomitant radiation [3, 7], so that the service items in the billing should be clear. However, the small number of patients who can be assigned suggests that many patients are treated off label. In agreement with the literature, and as per German Off-label allowance, it should be mentioned that carboplatin (in lung cancer licensed for SCLC patients only in Germany) can be prescribed off-label for NSCLC [2, 32–34]. In summary, no generally applicable algorithm for the selection of the patients concerned can currently be developed. However, it is expected that progress made in recent years towards personalized precision therapy for patients with lung cancer will allow a clearer assignment of patients based on documented billing data in the future.

In addition to drug-based therapy, surgical intervention is also a treatment option for lung cancer. In SCLC, this treatment approach is only recommended in stages I–II (very limited disease) [3, 6]. In NSCLC, by contrast, surgery can be part of the therapy at various stages [2, 6]. This explains the greater incidence of surgical intervention among NSCLC patients compared to SCLC patients. Overall, however, the number of patients with a documented surgical intervention was low, which is probably due to the fact, that many patients in the analysis population were only diagnosed at a late stage (i.e., with distant metastases, stage IV), when surgery is no longer indicated.

With regard to the diagnosis of lung cancer, the majority of the patients were initially coded during hospitalization. This is because around 90% of lung cancer patients initially show symptoms of the disease which are caused by the primary tumor and/or metastases already present [6]. After the first visit to the primary-care physician or pulmonologist, they are referred to a specialist center for further diagnostic clarification and confirmation of the suspected diagnosis. Since bronchoscopy is required for this purpose, and since this is usually done in an inpatient setting, the diagnosis is usually first coded in a hospital. With regard to the question of inpatient/outpatient care, a bias cannot be ruled out due to the hierarchy of the treating institutions applied in this analysis. For example, many pulmonologists working in the field of oncology are based in the specialist centers, so that although they make the diagnosis per se, in the present analysis “inpatient hospital” stays took precedence as the therapist. Due to the time-consuming diagnostics involved, a hospital stay of several days is usually required for those patients, which in turn explains the predominance of inpatient hospital stays in the initial coding compared to outpatient hospital stays.

Bronchoscopy is very important as part of the diagnosis itself [6, 35]. The majority of NSCLC and SCLC patients underwent bronchoscopy (NSCLC patients: 82.7%, SCLC patients: 80.8%). All bronchoscopies, regardless of their assignment to one of the cancer types, were also performed in a hospital setting. It was noteworthy in the present analysis that almost 78% of the unclassifiable patients and patients receiving “other therapy” did not undergo bronchoscopy. A reason why bronchoscopy was not documented for those patients can be that bronchoscopy in a study setting may be performed more frequently than in real world and thus be reimbursed by the sponsor of the study. Also, bronchoscopy is not required in the presence of distant metastases, e.g., in the liver, that have been histologically confirmed by liver biopsy. It should be noted that regarding determination of cancer type apart from the bronchoscopy, the present analysis only evaluated billing related to testing for biomarkers, so that it remains open whether there were other, possibly individual-related, reasons that supported lung cancer diagnosis and treatment for the unclassifiable patients.

It was noteworthy in the present analysis that biomarker testing was only documented for a small percentage of NSCLC patients (10.9% of NSCLC patients). This lack of data on biomarker test results is a limitation in the current analysis. Biomarker testing, e.g. for EGFR or ALK, is only recommended for non-squamous NSCLC, and differentiation from squamous NSCLC is not possible on the basis of the available data (see above) [2, 6, 35]. With regard to the treatment group, most of the biomarker tests were carried out in the “inhibitor therapy” group. This is understandable because the inhibitor is selected on the basis of the test results. Histopathological PD-L1 testing is not usually billed separately. Another reason for the poor testing rate may be due to the remuneration situation. Biomarker testing in a hospital setting is not reimbursed by the statutory health insurance funds. Another reason could also be that testing was carried out during the observation period in connection with large collaborative projects such as the Network Genomic Medicine Lung Cancer [36], or was billed via separate (integrated care) contracts, so that they were not included in the underlying data. The number of tests actually carried out could therefore be significantly higher than the results of the present analysis would indicate. This is also supported by the results of the German Lung Cancer Registry’s CRISP study (Clinical Research Platform into Molecular Testing, Treatment and Outcome of (Non)-Small Cell Lung Carcinoma Patients) [37]. The aim of CRISP is to collect valid and representative routine healthcare and quality-of-life data in order to reflect the current clinical reality of patients with metastatic NSCLC. Latest results have shown that around 83% of the lung cancer patients included in the cohort study (*n* = 2.204) were tested for EGFR, ALK, ROS-1, PD-L1 and / or BRAF [37].

The diagnosis of lung cancer usually has far-reaching consequences for those affected [38, 39]. Since the diagnosis is usually only made at an advanced or metastatic stage, the resulting limited prognosis requires rapid intervention. This involves hospital stays for diagnostic testing and treatment, visits to primary-care physicians and specialists, and visits to pharmacies. This results in a treatment-related impairment of freedom of the patient, which is added to the burden caused by the diagnosis itself. The present analysis shows that treatment-related impairment of freedom increased greatly in the index quarter as well as in the first quarter after diagnosis. In the index quarter, this is due largely to hospitalizations for diagnostic testing and subsequent treatment (surgery, chemotherapy, radiation). The number of visits to a specialist, however, increased significantly after the index quarter, regardless of the lung cancer type identified. This is also related to the treatment given. Both chemotherapy and radiotherapy are carried out on an outpatient basis in Germany, which has led to an increase in specialist visits. In Germany, oral cancer drugs, which are administered on an outpatient basis in a practice, are obtained by the patients themselves at a pharmacy. Consequently, there is a significant increase in pharmacy visits in the index quarter and in the following quarter, which is also reflected in the present analysis. With regard to cancer type, it was found that the time taken up by illness and treatment, especially for SCLC patients, is characterized by specialist visits. Because SCLC patients usually receive platinum-based chemotherapy [3, 6], and because this is carried out on several consecutive days per week, the number of specialist visits is particularly high for those patients. In the case of NSCLC, on the other hand, the time span between the individual infusions is several weeks (immunotherapeutic agents). Some of the drugs are also given orally (inhibitors). Thus, both therapeutic approaches require a specialist visit much less often [3].

Another aspect of needs-based care for lung cancer patients is the division of the supply infrastructure. In rural areas in Germany, the density of physicians is declining [40], which can lead to greater, treatment-related impairment of freedom compared to patients in the urban population. The present analysis shows that the majority of lung cancer patients live in urban areas. Besides smoking, various environmental factors are also responsible for the development of lung cancer. It is therefore suspected that the demonstrably higher exposure of people in urban areas to carcinogenic environmental factors may also lead to an increased number of lung cancer cases in urban areas [41]. However, the present analysis does not allow any valid conclusions to be drawn with regard to this question. In terms of treatment-related impairment of freedom, there were only marginal differences between patients in rural and urban areas. Similar results were also obtained in a recently published German study investigating whether the region of residence results in differences in the supportive care of lung cancer patients [42]. In the present analysis, only the number of specialist visits was significantly higher for SCLC patients in rural areas compared to those in urban areas. This may be due to age structure and associated comorbidities [43]. Overall, the treatment-related impairment of freedom is highest in the index quarter and in the following quarter irrespective of the cancer type and geographical distribution. This time period may therefore be the starting point for improving the current situation. Moreover, thanks to newer therapeutic approaches for the treatment of NSCLC, the number of specialist visits can be reduced, since drug administration is required less frequently in comparison to conventional chemotherapy.

## Conclusion

The present analysis confirms that the use of healthcare data on statutory health insurance patients is a valuable additional tool for evaluating needs-based care. It provides insights into the diagnostic measures taken and the treatments used based on the EBM and OPS codes and the ATC classification and thus models the real-world treatment situation. In addition, it was possible to determine where lung cancer is diagnosed and to identify the disease- and treatment-related impairment of freedom which the diagnosis imposes on patients. However, the present analysis also revealed weaknesses. The ICD-10 system currently in use for diagnostic purposes, for example, is not sufficient for differentiating between NSCLC and SCLC. Nevertheless, this is important due to the different treatment approaches and the different needs of those affected. Furthermore, some drugs licensed for a specific lung cancer type are used off-label for the other, which makes treatment-based assignment of patients to one of the two cancer types difficult. Moreover, the establishment of an appropriate selection algorithm has so far been stymied. Findings from retrospective analyzes of the healthcare data of the statutory health insurance funds, as used in the approach presented here, can currently only be used to a limited extent in decision-making with regard to the medical care of patients and as a basis for the allocation of resources. However, the data presented provide valuable information about current learning fields in the healthcare of patients suffering from lung cancer.

## Supplementary Information

**Additional file 1.**

## Data Availability

The datasets used and/or analysed during the current study available from the corresponding author on reasonable request. The MSD data sharing website (available at: http://engagezone.msd.com/ds_documentation.php) outlines the process and requirements for submitting a data request.
