# Supplementary material for: Use of algorithms for identifying patients in a German claims database: learnings from a lung cancer case
Source: BMC Health Serv Res. 2022 Jun 28;22:834. doi: 10.1186/s12913-022-07982-8 (PMC9241287; doi:10.1186/s12913-022-07982-8)
Supplement: Supplementary file 1 [file 12913_2022_7982_MOESM1_ESM.odt]

Supplements

Supplemental file 1: Cancer differentiation based on the ATC classification and OPS codes

The figure shows the drugs used along with the corresponding ATC code, any additional OPS code used, and the derived cancer type. (A) Coding algorithm used for classifying NSCLC and SCLC. (B) Proportion of patients who could be assigned to a cancer type based on the coded treatment.

a

| Name | ATC | OPS | Type |
| --- | --- | --- | --- |
| Pemetrexed | L01BA04 | 6-001.c | NSCLC |
| 5-Fluoruracil | L01BC02 |  | NSCLC |
| Gemcitabine | L01BC05 | 6-001.1 | NSCLC |
| Vinorelbine | L01CA04 |  | NSCLC |
| Nab-paclitaxel | L01CD01 | 6-005.d | NSCLC |
| Paclitaxel | L01CD01 | 6-001.f | NSCLC |
| Mitomycin | L01DC03 |  | NSCLC |
| Avelumab | L01X-C |  | NSCLC |
| Cetuximab | L01XC06 | 6-001.a | NSCLC |
| Bevacizumab | L01XC07 | 6-002.9 | NSCLC |
| Ipilimumab | L01XC11 | 6-006.h | NSCLC |
| Nivolumab | L01XC17 | 6-008.m | NSCLC |
| Pembrolizumab | L01XC18 | 6-009.3 | NSCLC |
| Necitumumab | L01XC22 | 6-009.g | NSCLC |
| Durvalumab | L01XC28 |  | NSCLC |
| Atezolizumab | L01XC32 |  | NSCLC |
| Gefitinib | L01XE02 |  | NSCLC |
| Erlotinib | L01XE03 |  | NSCLC |
| Afatinib | L01XE13 |  | NSCLC |
| Crizotinib | L01XE16 | 6-006.c | NSCLC |
| Dabrafenib | L01XE23 | 6-007.5 | NSCLC |
| Trametinib | L01XE25 | 6-009.7 | NSCLC |
| Ceritinib | L01XE28 | 6-008.a | NSCLC |
| Nintedanib | L01XE31 |  | NSCLC |
| Osimertinib | L01XE35 |  | NSCLC |
| Folic acid | V03AF03 |  | NSCLC |
| Cyclophosphamide | L01AA01 |  | SCLC |
| Ifosfamide | L01AA06 |  | SCLC |
| Trofosfamide | L01AA07 |  | SCLC |
| Lomustine | L01AD02 |  | SCLC |
| Etoposide | L01CB01 |  | SCLC |
| Vincristine | L01CA02 |  | SCLC |
| Doxorubicin | L01DB01 | 6-001.b | SCLC |
| Doxorubicin | L01DB01 | 6-002.8 | SCLC |
| Epirubicin | L01DB03 |  | SCLC |
| Carboplatin | L01XA02 |  | SCLC |
| Topotecan | L01XX17 | 6-002.4 | SCLC |
| Irinotecan | L01XX19 | 6-001.3 | SCLC |
| Liposomal irinotecan | L01XX19 | 6-009.e | SCLC |

b

Supplemental file 2: Coding to identify patients with surgical intervention

| Surgical intervention | Procedure |
| --- | --- |
| OPS 5-320 | Excision and destruction of diseased bronchial tissue |
| OPS 5-321 | Other bronchial excision and resection (without resection of the pulmonary parenchyma) |
| OPS 5-322 | Atypical lung resection |
| OPS 5-323 | Pulmonary segment resection or bisegment resection |
| OPS 5-324 | Simple pulmonary lobectomy or bilobectomy |
| OPS 5-325 | Extended pulmonary lobectomy or bilobectomy |
| OPS 5-327 | Simple (pleuro)pneumonectomy |
| OPS 5-328 | Extended (pleuro)pneumonectomy |
| OPS 5-329 | Other pulmonary and bronchial excisions |
| OPS 5-333 | Adhesiolysis of the lung and thoracic wall |
| OPS 5-339.2 | Destruction of diseased pulmonary tissue |
| OPS 5-342 | Excision and destruction of diseased mediastinal tissue |
| OPS 5-343 | Excision and destruction of (diseased) thoracic wall tissue |
| OPS 5-344 | Pleurectomy |
| OPS 5-349.6 | Repeat surgery of the lung, bronchus, thoracic wall, pleura |
| OPS 5-40 | Lymphatic tissue surgery |

OPS=German inpatient Operations and Procedure Code.

Supplemental file 3: Coding key to determine the discipline of the treating physician 

| Code | Definition |
| --- | --- |
| Hospital (inpatient)* | Hospital (inpatient, partially inpatient treatment with information on the specialist department) |
| Hospital outpatient | Hospital (outpatient treatment) |
| Oncologist | Outpatient treatment – specialist group code 27# |
| Pulmonologist | Outpatient treatment – specialist group code 30# |
| Radiologist | Outpatient treatment – specialist group code 62# or 65# |
| Internist | Outpatient treatment – specialist group code 23# |
| Primary-care physician | Outpatient treatment – specialist group code 01#, 02# or 03# |
| Other office-based physician | Outpatient treatment – other specialist group code |

* Hospital (inpatient) is the code element with the greatest weighting. # Specialist group code: numbers 8 and 9 of the lifelong physician’s number for SHI-accredited physicians.

Supplemental file 4:

(A) gives an overview of the therapeutic measures during second-line therapy, including those patients who could not be observed in line, for whom none of the defined therapies were documented, or who received second-line therapy (late therapy). The proportion of the total population who received or did not receive the respective therapy and/or measure is expressed in percent. (B) Comparison of the use of drug therapy and radiotherapy in relation to the classification group. The percent figures indicate the proportion of patients within the respective cancer-type population for whom the listed therapy was coded. n indicates the number of patients who were assigned to each cancer type or the group of unclassifiable patients. The patients were assigned to the respective therapies hierarchically. Note, the gap in the graph represents an axis break of the y-axis to allow for presenting all data in one single graph. (C) Percent of patients for whom a surgical intervention was coded, considering the cancer type and the coded first-line therapy. n indicates the number of patients who were assigned both to the respective cancer type and to the listed therapy based on the coding. B: bronchoscopy, OP: surgical intervention, n.c.: not classifiable.

Supplemental file 5: Treating institution or physician

The table shows the institution or physician that coded the diagnosis for each classification group in connection with second-line therapy. The percent figures express the proportion of patients coded by each “therapist” category. A hierarchy was defined to avoid patients being assigned to multiple categories (see description of Supplemental file 3).

| % | Hospital inpatient | Hospital outpatient | Other physician | Unknown | Total |
| --- | --- | --- | --- | --- | --- |
| NSCLC | 69.9 | 5.9 | 23.5 | 0.7 | 100.0 (100.0) |
| SCLC | 50.7 | 11.1 | 36.9 | 0.0 | 100.0 (98.7) |
| Unclass­ifiable | 72.9 | 3.8 | 21.8 | 1.5 | 100.0 (100.0) |

Supplemental file 6:

The diagnostic measures “bronchoscopy” (A) and “testing for biomarkers” (B) were analyzed concerning cancer type and the coded first-line therapy. (A) shows the percent of patients who had bronchoscopy (black bar, ■) or who did not have bronchoscopy (white bar, □) in relation to the coded first-line therapy within the cancer type (100% = all patients of the treatment group within the cancer classification group). (B) lists the biomarker testing of the incident patients. This shows the percent of patients for whom coded biomarker testing was carried out in connection with standard care within the treatment group of each cancer classification group (n = 100%), taking into account the respective coding therapist (hospital [H] vs. other office-based doctor) vs. no biomarker testing).
